# The effect of spontaneous osteoarthritis on conditioned pain modulation in the canine model

**DOI:** 10.1038/s41598-020-58499-1

**Published:** 2020-02-03

**Authors:** King Wa Chiu, Jon Hash, Rachel Meyers, B. Duncan X. Lascelles

**Affiliations:** 1 Translational Research in Pain, Comparative Pain Research and Education Centre, North Carolina State University College of Veterinary Medicine, Raleigh, NC United States; 2 Comparative Medicine Institute, North Carolina State University, Raleigh, NC United States; 3 Center for Pain Research and Innovation, University of North Carolina School of Dentistry, Chapel Hill, NC United States; 4 Center for Translational Pain Medicine, Department of Anesthesiology, Duke University, Durham, NC United States

**Keywords:** Biomarkers, Musculoskeletal system

## Abstract

Endogenous Pain Modulation (EPM) impairment is a significant contributor to chronic pain. Conditioned pain modulation (CPM) testing assesses EPM function. Osteoarthritic (OA) dogs are good translational models, but CPM has not been explored. Our aim was to assess EPM impairment in OA dogs compared to controls using CPM. We hypothesized that CPM testing would demonstrate EPM impairment in OA dogs compared to controls. Dogs with stifle/hip OA and demographically-matched controls were recruited. The pre-conditioning test stimulus, using mechanical/thermal quantitative sensory testing (MQST or TQST), were performed at the metatarsus. A 22N blunt probe (conditioning stimulus) was applied to the contralateral antebrachium for 2 minutes, followed by MQST or TQST (post-conditioning test stimulus). The threshold changes from pre to post-conditioning (∆MQST and ∆TQST) were compared between OA and control dogs. Twenty-four client-owned dogs (OA, n = 11; controls, n = 13) were recruited. The ∆MQST(p < 0.001) and ∆TQST(p < 0.001) increased in control dogs but not OA dogs (∆MQST p = 0.65; ∆TQST p = 0.76). Both ∆MQST(p < 0.001) and ∆TQST(p < 0.001) were different between the OA and control groups. These are the first data showing that EPM impairment is associated with canine OA pain. The spontaneous OA dog model may be used to test drugs that normalize EPM function.

## Introduction

In the US, persistent pain affects one-third of the human population with an economic impact of US$600 billion each year, which is more than cardiovascular disease and cancer combined^1^. Osteoarthritis (OA) is a major contributor to persistent pain and is one of the top causes of disability in both the US and the UK^2^. In humans, Endogenous Pain Modulation (EPM) – the ability of the body to control noxious input to the central nervous system - been shown to be deficient in patients suffering from numerous chronic pain condition, including OA^3^. EPM is determined by the balance between descending inhibition and descending facilitation. The EPM system appears to be mediated by the caudal medulla of subnucleaus reticularis dorsalis, and the rostral ventral medulla, and is activated by ascending noxious stimuli^4^.

Patients with an impaired EPM may have increased pain sensitivity, which contributes to the persistent pain state^5^. The degree of EPM impairment varies between patients with the same disease. Studies have shown that human patients suffering from hip or knee OA have different levels of endogenous pain modulation (EPM) impairment, which contributes to the heterogeneity of pain mechanisms^3,6^.

To evaluate the EPM using the CPM paradigm, quantitative sensory testing (QST) is performed, which can in the form of a mechanical (MQST) or thermal stimulus (TQST) test stimulus. A test stimulus is applied in the absence (pre-conditioning), and the presence (post-conditioning) of a noxious stimulus applied to a remote body region. The change in pain threshold from pre- to post-conditioning (∆MQST and ∆TQST) is higher in healthy controls compared with patients with impaired EPM^7^. The different levels of EPM impairment between OA patients may explain their difference in pain level as well as variation in treatment response^8–10^.

Naturally occurring OA in dogs is similar to that of human OA biomechanically, structurally, histologically, and molecularly, and has been used as a translational model for studying human OA pain^11–14^. Previously, our research group showed that dogs with spontaneous OA pain have central sensitization as evaluated by QST, which is similar to human pains with OA pain^10^. Graven-Nielsen and colleagues performed CPM testing in human patients with severely painful knee OA with the MQST stimulus applied to the peripatellar region, lower leg, and forearm. Ischemic compression of the forearm was used as a conditioning stimulus. Human patients with knee OA had significantly lower post-conditioning MQST compared with healthy controls^6^. Arendt-Nielsen *et al*.^15^ also showed that patients with knee OA did not show a significant change from pre- to post-conditioning MQST (applied to the peripatellar region, tibialis anterior, and extensor carpi radialis longus muscle), whereas healthy controls had a significant increase of MQST after the application of the noxious stimulus (ischemic compression at the contralateral forearm)^3^. However, the use of CPM to evaluate EPM has not been studied in dogs with OA.

This study aimed to compare EPM impairment in OA dogs compared to healthy controls using CPM testing. The hypothesis was dogs with spontaneous OA would show EPM impairment based on CPM testing compared to controls.

## Results

Twenty-four client-owned dogs (OA affected, n = 11; and controls, n = 13) were recruited. The demographics are shown in Table 1. The nonparametric variables included weight, Liverpool Osteoarthritis in Dogs (LOAD) score, ∆TQST, pre-conditioning and post-conditioning data of both MQST and TQST. The mean age in the OA and control groups were 7.6 ± 3.0 and 4.5 ± 2.7 years old respectively. The mean body weight of the OA group and control groups were 30.8 ± 8.8 kg and 24.5 ± 5.1 kg respectively. In the OA group, the right hindlimb and left hindlimb were primarily affected (and tested) in six and five dogs respectively. In the control group, the right hindlimb and left hindlimb were tested in seven and six dogs respectively. The patient age (p = 0.01) was significantly different between the groups, with the mean difference being 3.2 ± 1.2 years old. Weight (p = 0.05), breed (p = 0.09), sex (p = 0.14), and testing site (p = 0.93) were not different across groups. The LOAD score in OA dogs (21, 9–29) was higher than control dogs (3, 0–7) (p < 0.001).

**Table 1:** Patient demographics of recruited OA and control dogs.

	OA (n = 11)	Control (n = 13)	All (n = 26)
Age			
Mean	7.6	4.5	5.9
Median	8	4	5.5
Range	2–12	1–9	1–12
SD	3.0	2.8	3.3
SEM	0.9	0.8	0.7
Sex			
Male castrated	2	2	4
Male intact		3	3
Female spayed	9	6	15
Female intact		2	2
Body weight			
Mean	30.8	24.5	27.4
Median	29.4	22.6	26.6
Range	19.5–48.7	18.2–31.7	18.2–48.7
SD	8.8	5.1	7.6
SEM	2.6	1.42	1.5
Breed			
American Foxhound		1	1
Australian Shepherd		1	1
Border Collie	1		1
German Shephard Dog	3		3
Golden Retriever	1	4	5
Hound	1		1
Labrador Retriever	1	4	5
Mixed breed dog	1	2	3
Portuguese Water dog		1	1
Staffordshire Bull Terrier	3		3
LOAD Score			
Mean	19.7	2.7	10.8
Median	21	3	7
Range	9–29	0–7	0–29
SD	8.0	2.4	10.3
SEM	2.4	0.7	2.2
Total pain score of the painful limb			
Mean	3.5	0	1.7
Median	3	0	0
Range	3–6	0	0–6
SD	1.3	0	2.1
SEM	0.4	0	0.4

The pre-conditioning and post-conditioning MQST and TQST threshold values and the deltas are detailed in Tables 2 and 3. In the control group, the mean pre-conditioning MQST threshold ± SD was 1299 ± 745 g, and the mean post-conditioning MQST threshold was 1967 ± 943 g. The mean ∆MQST was 668 ± 288 g. The mean pre-conditioning TQST threshold was 8.0 ± 5.8 s and mean post-conditioning TQST threshold was 14.9 ± 6.1 s. The mean ∆TQST was 6.9 ± 4.4 s. A significant increase in threshold was noted in both MQST (p < 0.001) and TQST (p < 0.001) during the application of the noxious stimulus.

**Table 2:** Descriptive statistics of pre-conditioning, post-conditioning, ∆ and MQST threshold before and during application of the noxious stimulus.

	OA (n = 11) (g)			Control (n = 13) (g)		
	Pre-conditioning	Post-conditioning	∆	Pre-conditioning	Post-conditioning	∆
Mean	1208	1158	−50	1299	1967	668
Range	545–1888	724–1832	−441–179	428–3295	879–4410	241–1115
SD	454	385	216	745	943	288
SEM	137	116	65	206	261	80

**Table 3:** Summary of TQST threshold before and during application of the noxious stimulus.

	OA (n = 11) (s)			Control (n = 13) (s)		
	Pre-conditioning	Post-conditioning	∆	Pre-conditioning	Post-conditioning	∆
Mean	11.4	11.5	0.1	8.01	14.89	6.9
Range	2.7–20	2.12–20	0	2.16–20	3.20–20	0–14.6
SD	6.6	6.9	0.9	5.75	6.11	4.4
SEM	2.0	2.1	0.2	1.59	1.69	1.3

In the OA group, the mean pre-conditioning mechanical QST threshold ± SD was 1207 ± 454 g, and the mean post-conditioning MQST threshold was 1158 ± 385 g. The mean ∆MQST was -50 ± 215 g. The mean pre-conditioning TQST threshold was 11.4 ± 6.6 s and mean post-conditioning TQST threshold was 11.5 ± 6.9 s. The mean ∆TQST was 0.1 ± 0.9 s. No significant change in threshold was found in both MQST (p = 0.65) and TQST (p = 0.76) during the application of the noxious stimulus.

There were significant differences in ∆MQST (717 ± 103 g; p < 0.001) and ∆TQST (6.7 ± 1.3 s; p = 0.002) between OA and control dogs. Age and OA status were included in the logistic regression model to assess their effect on both ∆MQST and ∆TQST. The effect of OA status on both ∆MQST (p < 0.001) and ∆TQST (p < 0.001) was statistically significant. The effect of age on both ∆MQST (p = 0.50) and ∆TQST (p = 0.08) was statistically insignificant. The covariate statistics of the model for ∆MQST and ∆TQST are shown in Tables 4 and 5.

**Table 4:** Estimates and CIs for the covariates tested in the logistic regression model on ∆MQST.

	Estimate	CI_95_	SE	p-value
Intercept	229	−37 to 495	128	0.09
Age	13	−27 to 53	19	0.50
OA status	380	252 to 508	62	<0.001

**Table 5:** Covariates statistics of the logistic regression model on ∆TQST.

	Estimate	SE	p-value	CI_95_
Intercept	0.95	1.55	0.54	−2.27 to 4.17
Age	0.42	0.23	0.08	−0.06 to 0.90
OA status	4.05	0.74	<0.001	2.50 to 5.59

## Discussion

Both MQST and TQST post-conditioning threshold increased significantly in control dogs in response to the conditioning stimulus, but not in dogs with spontaneous OA. The results of this study suggest that EPM is impaired in dogs with spontaneous osteoarthritis. These findings are similar to those in humans. Both Graven-Nielsen *et al*.^15^ and Arendt-Nielsen *et al*.^16^ showed human OA patients have less efficient CPM compared with healthy controls as assessed using an MQST stimulus^3,6^. In contrast, in humans, the use of TQST to assess CPM has not produced such consistent results. Valencia *et al*.^18^ and King *et al*.^19^ used TQST as a test stimulus for CPM testing^18,19^. Valencia and colleagues compared CPM in patients with and without shoulder pain and demonstrated a larger delta in those with shoulder pain compared to controls. However, the study by King *et al*.^19^ did not show a significant difference in CPM between the control and knee OA group. The inconsistent results may be due to the reliability of TQST to assess CPM. Granovsky *et al*. (2016) showed that the standard parallel TQST CPM paradigm had poor to fair intraclass correlation (ICC = 0.34). However, it should be noted that the conditioning and test stimulus used in the three TQST CPM studies cited were different. For the conditioning stimulus, Granovsky *et al*.^19^ used a hot-water bath whereas both Valencia *et al*.^18^ and King *et al*.^19^ used a cold-water bath. Granovsky *et al*.^19^, Valencia *et al*.^18^ and King *et al*.^19^ also used different testing protocols. We have not yet tested the repeatability of CPM testing in the canine spontaneous OA model, nor have we comprehensively evaluated different conditioning stimuli.

To the authors’ knowledge, this is also the first study utilizing a mechanical actuator to induce a conditioning stimulus for CPM assessment. Le Bars *et al*.^20^ studied diffuse noxious inhibitory control (DNIC) in rats (currently referred to as EPM in humans and canines) by pinching the tail to induce a conditioning stimulus^20^. Using this methodology, DNIC was activated in rats. One of the advantages of using the mechanical actuator in dogs is its feasibility. Subjects tested are not required to perform any activities to facilitate the induction of the noxious stimulus. In human studies, using a pressure cuff to induce a noxious stimulus requires patients to repeat a handgrip ten times or more to create a painful ischemic effect^17^. In our early pilot work (see Supplementary Materials) of investigating the right conditioning stimulus, we applied a pressure cuff at the distal antebrachium contralateral to the affected limb. The cuff was inflated to 200 mmHg of pressure. The dogs were encouraged to walk on a lead for 2 minutes to mimic the handgrip to induce an ischemic state. Many of the dogs were not willing to walk and did not show any signs of pain (e.g., mydriasis, tachycardia, limb withdrawal and vocalization) towards the pressure cuff. It was questionable whether those dogs experienced a noxious stimulus. Our pilot work indicated that a consistent noxious stimulus was achieved by using the pneumatic limb actuator and all dogs showed signs of discomfort towards the conditioning stimulus. Another method to induce a noxious stimulus in humans is by immersing the patient’s hand into painful hot water or iced water^9,21^. The pilot work in our laboratory showed that using ice water immersion was not a sufficient conditioning stimulus for both sedated and anesthetized dogs when using nociceptive withdrawal testing and EMG measures to assess CPM (submitted for publication). The skin temperature of the interdigital skin dropped to 4.9 °C 10 minutes after immersing the carpus into ice water, but despite maintaining the paw in the ice water bath, the skin temperature increased subsequently. This inability to cool the limb may explain the failure of this conditioning stimulus. Other methods to induce a conditioning stimulus in human pain research include a hot water bath, thermal contact-heat, and cold pressor test. The optimal methodology of inducing a conditioning stimulus in the canine model warrants further investigation.

Control dogs were recruited with the intention of having similar demographics. The age of the OA group was significantly higher than that of the control group. This finding, however, was not unexpected. Anderson *et al*.^22^ collected data from 4196 dogs diagnosed with OA and age were shown to be significant risk factors for OA^22^. CPM studies in humans suggested an age effect on endogenous pain modulation^23,24^. Although these studies excluded patients with chronic pain conditions based on history, no clinical exam or diagnostics were performed to screen for chronic pain conditions before inclusion. Underlying chronic painful conditions hence could still be a confounding factor in these studies due to its prevalence in the senior population^25^. In our study, the effect of age was shown to be statistically insignificant based on our logistic regression model. However, further work to evaluate the effect of age on CPM in this canine model should be performed.

In this study, EPM was shown to be impaired in dogs with spontaneous OA, similar to humans. The naturally occurring OA canine model could, therefore, be used to test drugs that may reverse EPM impairment. To further validate this model, future studies should investigate the use of duloxetine or tapentadol to normalize EPM in dogs. Studies on the repeatability of CPM testing should also be performed to assess the reliability of EPM evaluation in dogs.

We have demonstrated, for the first time in client-owned dogs, that EPM is impaired by chronic spontaneous OA as compared with healthy control dogs. The results of this study parallel the findings in humans with OA, which supports the use of the canine model to study medications that normalizes EPM. Further studies should assess the clinical consequences of EPM impairment and investigate the type of treatments that can reverse EPM impairment in both humans and dogs.

## Methods

This study was approved by North Carolina State University Institutional Animal Care and Use Committee (protocol no. 16-186-O). All the methods were performed in accordance with the relevant guidelines and regulations. The study was explained to all owners verbally and written informed consents were signed. Dogs enrolled in the study were all client-owned animals with spontaneous chronic (greater than 6 months) OA and associated pain. Healthy controls with a similar phenotype were recruited. The dogs of both groups were recruited over a 3-month period using email advertisements within the veterinary college, NC State community, and social media advertisements.

Dogs recruited were required to be over one year old and weigh ≥15 kg. Physical examination, including body condition score (BCS), orthopedic, and neurological examination was performed during the screening. The orthopedic exam was performed by K.W.C trained by B.D.X.L. Gait (lameness, stiffness, and posture), response to joint manipulation (range of motion, pain, crepitus, effusion, and thickening), and the degree of muscle atrophy were evaluated (pain score 0: does not notice manipulation; 4: Tries to escape or prevent from manipulation). Complete blood cell count, chemistry panel, and urinalysis were performed in all dogs to screen for other underlying diseases besides OA. A validated, owner-completed clinical metrology instrument – Liverpool Osteoarthritis in Dogs (LOAD) - was also utilized to assess the level of mobility impairment^18,19^. This questionnaire consists of 13 items with all items reported on a 5-point Likert-type scale, scored between 0 and 4. These scores are then summed to give an overall instrument score. Radiographs of clinically painful appendicular joints were taken under sedation with orthogonal views. A veterinary board-certified radiologist confirmed the radiographic findings. A combination of dexmedetomidine (0.003 mg/kg; Dexdomitor; Zoetis, NJ, USA) and hydromorphone (0.05 mg/kg; Hydromorphone Hydrochloride Injection, USP; West-Ward Pharmaceutical Corp. NJ, USA) was administered intravenously for sedation. The dexmedetomidine was reversed with atipamezole (0.03 mg/kg; Antisedan; Zoetis) administered intramuscularly.

Control dogs were required to have no history of impairment recognized by the owner (LOAD score < 7), no abnormalities detected on orthopedic (no pain, no decreased muscle mass, no joint instability or other pathology), and neurologic examinations. These dogs were not receiving any analgesics. Control dogs were required to have no evidence of abnormalities on clinical exam.

OA dogs were required to have a 6-month history of impaired mobility (LOAD score > 9) as reported by the owner. The site of OA was required to be at either the hip or stifle joint. Dogs with evidence of concurrent diseases other than OA based on clinical exam, bloodwork, and history were excluded from the study. OA dogs were required to be off analgesics for more than three weeks and steroids for more than four weeks. Only dogs with radiographic evidence of OA were included in the OA group.

### CPM testing

Procedures were performed in the Gait Laboratory, which is a 15 × 4 m separate room with two doors and no windows, located at the NCSU College of Veterinary Medicine. Dogs were given 5 minutes to acclimatize to the room and were then placed in lateral recumbency with minimal restraint by a male technician (J.H.) on a standard yoga mat. Dogs that required testing on the right pelvic limb were placed in left lateral recumbency and vice versa. When dogs were distracted by noises outside of the gait laboratory, such as construction and student leaving for lectures, data collection was stopped and attempted again after the distraction was ceased. Occasionally, dogs were rewarded with food and fresh water was available ad libitum.

**Figure 1:**
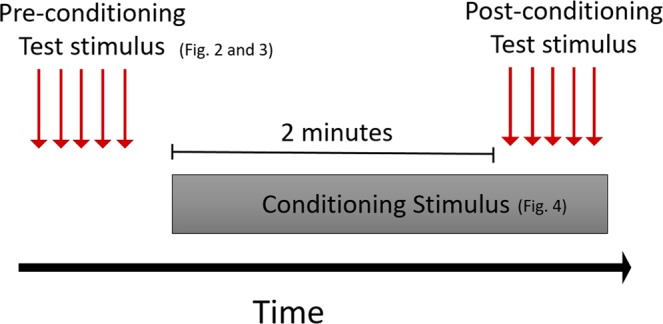
CPM testing paradigm illustration with the timeline of pre-conditioning, conditioning, and post-conditioning stimulus application.

CPM testing was performed by a male veterinarian (K.W.C.) trained by B.D.X.L. CPM was assessed using mechanical (MQST) and hot thermal (TQST) test stimulus in a randomized order. The order was generated using a computer program. Dogs were given a seven-minute break transitioning between test modalities. A minimum of 10 seconds was given in between test stimuli. The pre- and post-conditioning stimuli were defined as the test stimulus performed before and during application of the noxious stimulus respectively (Fig. 1). Delta (∆) was defined as the difference between pre- and post-conditioning threshold.

The test stimulus was applied to the OA affected leg or a randomized hindlimb at the dorsal surface of the metatarsus between metatarsal bones III and IV^20^. For both pre- and post-conditioning testing, five trials of the test stimulus was applied. Each testing site was moved a few millimeters away from the previously tested spot after each trial. The definition of the endpoint for both thermal and mechanical stimuli was a behavioral response indicative of a conscious perception of the stimulus: movement of the limb away from the probe with conscious perception of the stimulus, turning the head to look directly at the site, vocalization, or other consistent, clearly recognizable body movement indicating perception of the stimulus^20^ (Supplementary Data: https://bit.ly/2WCtI14)^20^. Simple reflex movement (such as twitching) was not considered an endpoint. It was on a 0–5 scale with 0 indicating no problem in collecting data and 5 indicating data were impossible to obtain.

#### MQST

**Figure 2:**
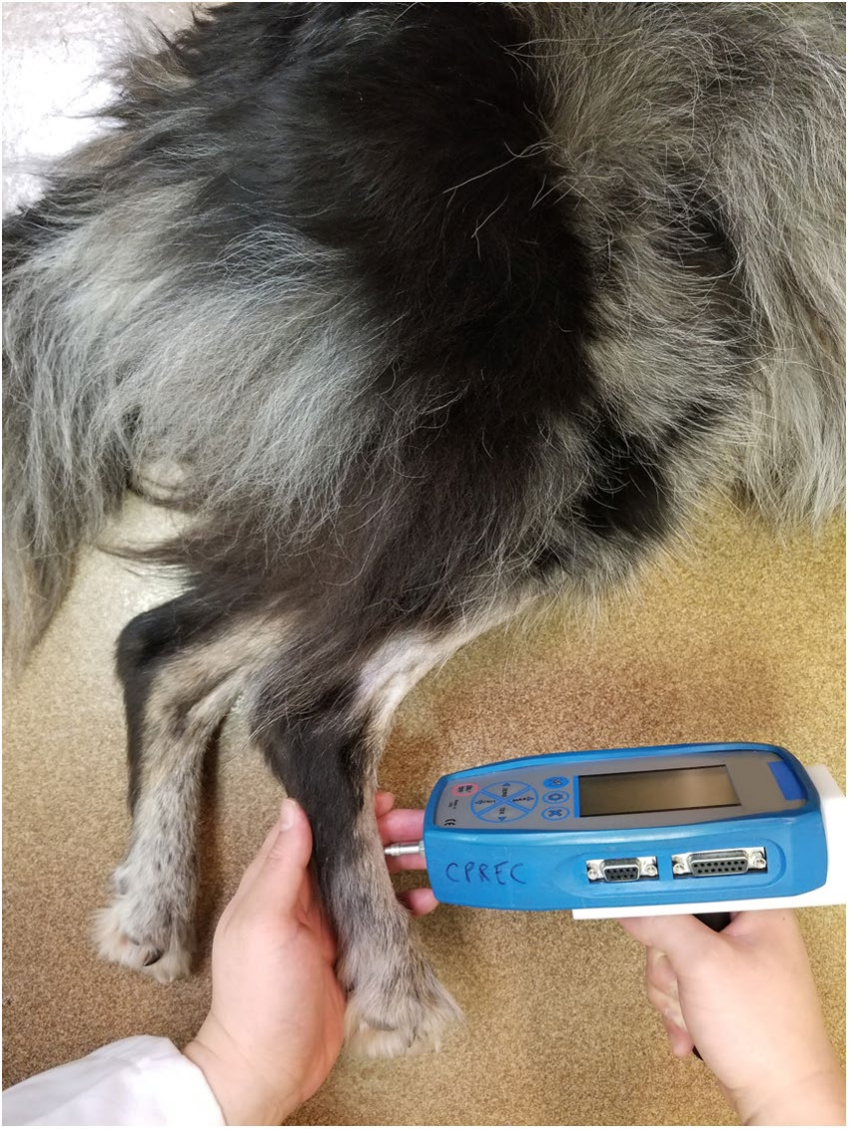
Application of the mechanical test stimulus using the Bioseb algometer.

MQST was performed using a blunt probe pressure algometer (SMALGO algometer; Bioseb, Vitrolles, France) (Fig. 2). The algometer has a seven mm^2^ flat diameter tip attached to a recording unit. The maximum force applied was 2500 g.

#### TQST

**Figure 3:**
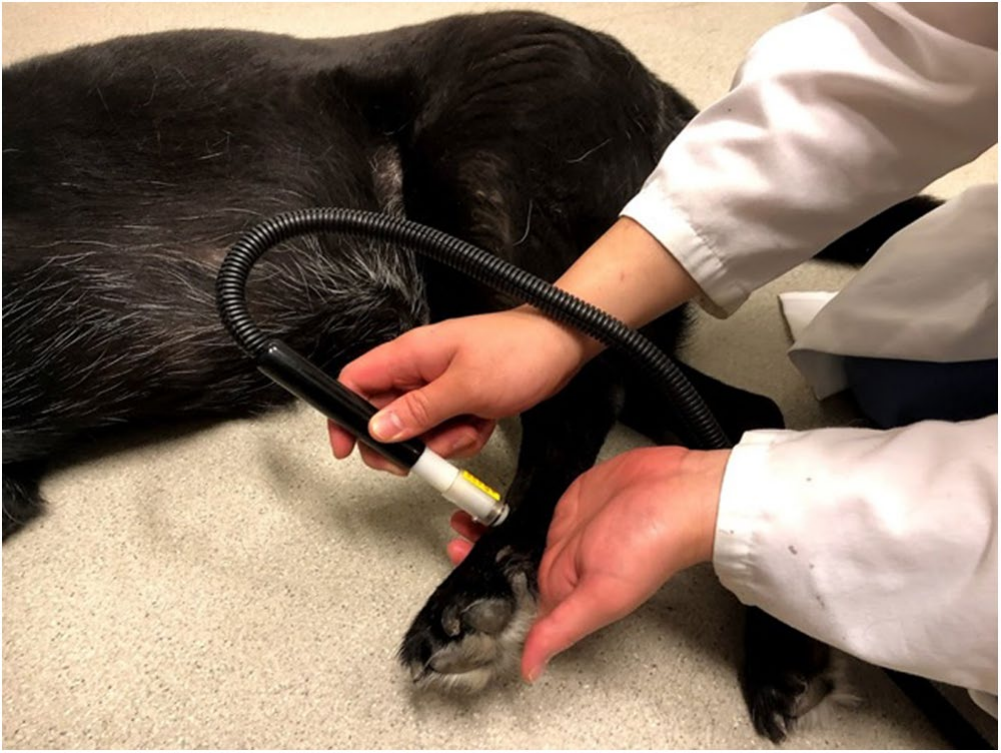
Application of the thermal test stimulus using the Physitemp NTE-2A at 49 °C.

The TQST device (NTE-2A; Physitemp Instrument, Clifton, NJ) had a 13 mm tip connected to a recirculating pump with a combined reservoir that has digital temperature control (Fig. 3). The temperature of the probe can be altered between 0–50 °C, maintained to within 0.1 °C. It delivered a thermal stimulus for a maximum of 20 s using a probe at 49 °C.

#### Conditioning stimulus

**Figure 4:**
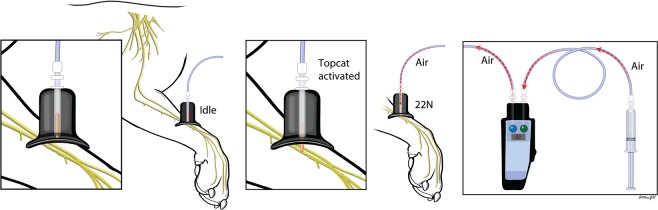
Application of the 22N mechanical conditioning stimulus to the antebrachium.

A noxious stimulus (conditioning stimulus) was generated using an algometer connected to a pneumatic limb actuator (ProdPro, Topcat Metrology Ltd, UK) with a 2.5 mm probe (Fig. 4)^16^. The actuator was applied to the medial aspect of the distal antebrachium contralateral to the hindlimb tested using a mounting boot. A force of 22N was applied using the actuator for two minutes before post-conditioning QST testing. The noxious nature of the stimulus was confirmed by observing clear behavioral indicators of discomfort, such as mydriasis, tachycardia, limb withdrawal, and vocalization. Our laboratory had previously attempted to use an ischemic cuff at 200 mmHg and ice water submersion to induce a conditioning stimulus at the contralateral forelimb. However, the noxious stimulus did not give reliable results.

#### Statistical analysis

The distribution of the data was assessed by the Shapiro-Wilk test. The t-test and Mann-Whitney U test were used to test parametric and non-parametric data respectively. The Pearson’s Chi-Square test was used to compare categorical variables between OA and control groups. A logistic regression model was used to address the effects of covariates on both ∆MQST and ∆TQST. Statistical analysis was performed by JMP (SAS, Raleigh, NC).

## Supplementary information

Condition pain modulation evaluation pilot data.

Video 1.

## Data Availability

The datasets generated during and/or analyzed during the current study are available from the corresponding author on reasonable request.
