# Supplementary material for: The effect of spontaneous osteoarthritis on conditioned pain modulation in the canine model
Source: Sci Rep. 2020 Feb 3;10:1694. doi: 10.1038/s41598-020-58499-1 (PMC6997173; doi:10.1038/s41598-020-58499-1)
Supplement: Supplementary file 1 — Condition pain modulation evaluation pilot data. [file 41598_2020_58499_MOESM1_ESM.docx]

**Title:** **The effect of spontaneous osteoarthritis on conditioned pain modulation in the canine model**

**Authors**: King Wa Chiu^1^, Jon Hash^1^, Rachel Meyers^1^, B. Duncan X. Lascelles*^1, 2, 3, 4^

**Affiliations**: 1. Translational Research in Pain, Comparative Pain Research and Education Centre, North Carolina State University College of Veterinary Medicine, Raleigh, NC, United States. 2. Comparative Medicine Institute, North Carolina State University, Raleigh, NC, United States. 3. Center for Pain Research and Innovation, University of North Carolina School of Dentistry, Chapel Hill, NC, United States. 4. Center for Translational Pain Medicine, Department of Anesthesiology, Duke University, Durham, NC

*Correspondence to dxlascel@ncsu.edu

**Supplementary material – Condition pain modulation evaluation pilot data**

Ischemic model for condition pain modulation evaluation (pilot work performed in present study)

The pre-conditioning testing was as described in the manuscript. A blood pressure cuff was at the distal antebrachium contralateral to the affected hindlimb. The cuff was inflated to 220 mmHg was used as a conditioning stimulus. The dog was encouraged to walk for two minutes while cuff was inflated. The post-conditioning testing was performed while the cuff was still inflated after two minutes as described in the manuscript. A total of two control dogs were recruited to evaluate the ischemic model for conditioned pain modulation evaluation. These dogs were five and eight years old, female spayed, Rottweiler and mixed breed dog respectively. After the cuff was inflated, both dogs did not display signs of discomfort, for example, mydriasis, withdrawal of leg, and vocalization. When the dogs attempted to walk, the blood pressure cuff reading changed due to movement of the leg. The pressure of the cuff also decreased after walking. The average pre-conditioning mechanical threshold were 1180 grams and 1099 grams. The average post-conditioning mechanical threshold were 1111 grams and 887 grams. The average delta mechanical threshold were -68 grams and -211 grams. The average pre-conditioning thermal threshold were 8.0 seconds and 20.0 seconds. The average post-conditioning thermal threshold were 3.0 seconds and 17.3 seconds. The average delta thermal threshold were -5 seconds and -2.5 seconds. Considering that the cuff pressure was highly variable and that the stimulus did not elicit a painful response, the authors decided not to use this model for testing.

Ice water immersion model for condition pain modulation evaluation (data submitted for publication)

Control dogs were sedated with acepromazine, sedated with alfaxalone or anesthetized with alfaxalone. An electrical test stimulus was used and was assessed by electromyographic. The conditioning stimulus was applied by ice water immersion of the forelimb. The interdigital skin temperature decreased in all three groups reaching the nadir (4.9-13.6^o^C) at 10 minutes after immersion. In the alfaxalone sedated group, skin temperatures were significantly higher (p=0.018) than the acepromazine sedated dogs. The skin temperature of both the alfaxalone sedated and anesthetized group returned to baseline within 10 minutes after removing the limb from the ice water, whereas the acepromazine sedated dogs still had significantly decreased temperature 10 minutes after discontinuation of ice water immersion (p=0.023). electromyographic magnitude no changes from pre- to post-conditioning, suggesting the endogenous pain modulation was not activated.
